# Optimization process of coffee pulp wines combined with the artificial neural network and response surface methodology

**DOI:** 10.1038/s41598-025-00147-7

**Published:** 2025-05-14

**Authors:** Rongsuo Hu, Fei Xu, Liyan Zhao, Wenjiang Dong

**Affiliations:** 1 College of Food and Technology, Nanjing Agriculture University, Nanjing, 210095 Jiangsu China; 2 Spice and Beverage Research Institute, Chinese Academy of Tropical Agricultural Sciences (CATAS), Wanning, 571533 Hainan China; 3 Key Laboratory of Processing Suitability and Quality Control of the Special Tropical Crops of Hainan Province, Wanning, 571533 Hainan China; 4 College of Food Science and Technology, Nanjing Agricultural University, Nanjing, 210095 Jiangsu China

**Keywords:** Coffee pulp wine, Process optimization, RSM, ANN-GA, Applied microbiology, Industrial microbiology

## Abstract

Coffee pulp wine was made from coffee pulp. The level range of fermentation factors was determined by one-factor experiment. The significance factors affecting fermentation were screened by Plackett-Burman and steepest climbing experiments, which were material-liquid ratio, initial pH, initial sugar and yeast amount, respectively. The screened factors were then subjected to a central combination design, and the results were optimized using RSM and ANN-GA. The ANN-GA shows a more accurate optimization effect compared with RSM and a higher degree of model fitting. The coefficient of determination (R^2^) of the ANN-GA predicted value was 0.9140, while the RMSE was 0.0896. The best results of optimization process showed that the material-liquid ratio was 4.25 : 95.75, the initial pH value was 6.92, the initial sugar concentration was 22.248%, the yeast addition was 1.98%, and the final predicted value was 10.255 mg/L. The research results provided a technical reference for the production of coffee pulp wines.

**Supplementary Information:**

The online version contains supplementary material available at 10.1038/s41598-025-00147-7.

## Introduction

Coffee pulp was a general term for the ectocarp and some pectin, accounting for about 45% of coffee fruits^1^. Coffee pulp was usually considered to be a by-product in the primary processing of coffee origin. In addition to a small amount of coffee pulps returned to the field as fertilizer, a large amount of coffee pulps were discarded as waste^2^. The high sugar concentration in coffee pulp result in the produce of unpleasant odor and wastewater during the process of fermentation and degradation. It may lead to the acidification of soil and rivers, seriously threatening the ecological environment and water system^2,3^.

Coffee pulp holds promising potential for advancement and utilization in relation to its nutrient composition and biological properties. Coffee pulp rich in carbohydrates, minerals, protein and so on^4^. It also contains a large number of bioactive ingredients such as anthocyanins, phenolic compounds and flavanols^5^, which have different preventive and therapeutic effects on chronic diseases^6^. Moreover, coffee pulp can be incorporated into various food items owing to its inherent sweetness^7^, which also be used as a raw material for fruit wine fermentation^2^. Therefore, coffee pulp had been regarded as a new type of food raw material in the food industry^8^.

The wine fermentation is a complex system, which is influenced by many factors. Process optimization research is the basis of wine fermentation, because it is a necessary link of the fermentation process. Although the research technology is relatively mature, the fermentation process optimization research of longan wine^9^, Chinese rice wine^10^, mulberries wine^11^ and sugarcane-papaya wine^12^ and other wines has been reported in succession in recent years. Coffee pulp wine was produced from the pulp and pectin, which was also known as fermented alcoholic drinks in some areas. Dry white wine was produced by using the coffee pulp in Central America^13^.

There are also some recent results on coffee pulp wines. Einfalt et al.^14^reported the process analysis and sensory evaluation of coffee pulp wine, which concluded that the ethanol yield was 8.26% (w/w). After sensory evaluation, it was concluded that plants and nuts are the most important terms to describe the perception of coffee peel wine. Kc et al.^15^reported the use of coffee pulp and pectin to develop alcoholic beverages, explored the fermentation process, chemical parameters and sensory characteristics of alcoholic beverages, and found that fermented alcoholic beverages had good taste and could be used for the development of alcoholic beverages. Bae et al.^16^ studied the use of coffee pulp as the starting material for the manufacture of alcoholic beverages (coffee pulp wine) and the yeast fermentation capacity, and evaluated the antioxidant activity of five yeast fermented coffee pulp wine. Hu et al.^2^ reported the mixed fermentation of four *S. cerevisiaes* to produce coffee pulp wine and the comparative evaluation of its flavor and sensory properties. Although there were some research results, the study of the fermentation process had not yet been reported.

Response surface methodology (RSM) is a set of mathematical techniques that describe the relation between several independent variables and one or more responses^17^, which is the most commonly used optimization method for the fermentation process. In recent years, some scholars have also tried to use other algorithms to optimize the fermentation process. The extraction process is optimized based on response surface analysis and reverse propagation neural network-genetic algorithm^18^. Dragoi et al. proposed and tested an improved, simple and flexible adaptive differential evolution algorithm for neural model to optimize the aerobic fermentation process through accurate modeling^19^.

Artificial neural networks (ANN) is a deep learning technique that can be used to model complex nonlinear relationships between variables^20^. Genetic algorithms (GA) is an efficient optimization technique that mimics biological evolutionary systems, such as selection, crossover, and mutation^21^. Over the past decade, ANN coupled with GA has been recently employed for optimization of fermentation processes and extraction conditions since it can help to find the optimum conditions with less effort^22^. For example, pectin extraction from sunflower seeds^23^, polyphenols extraction from green tea^24^, polyphenols extraction from pitaya skin^25^ and ellagittannin extraction from black raspberry seeds^26^. It may be that ANN can be used for modeling complex non-linear relationships between variables, more accurate than other fitting methods such as the RSM approach^20^.

This study aims to achieve the following goals: (I) The Plackett-Burman (PB) experiment and the steepest climbing experiment were used to determine the significant influencing factors, (II) Model establishment of ANN-GA and RSM, (III) Contrast the RSM and ANN-GA to determine the optimization method, and the optimal fermentation parameters. The research results can not only provide technical support for production, but also provide a reference for fermentation optimization.

## Materials and methods

### Sample collection and processing

The experimental raw material was Arabica coffee pulp, which was collected from Munaihe Industrial Park, Simao District, Pu’er City, Yunnan Province. The sampling work was assisted by the College of Tropical Crops, Yunnan Agricultural University. Coffee pulp was prepared by wet processing of coffee cherry and contains approximately 50% pectin of the total component^2^. The four species of *S. cerevisiaes* used in this experiment were purchased from the China Center of Industrial Culture Collection (CCIC), and their serial numbers were CICC1425, CICC1793, CICC1557, and CICC32762. The four strains were activated and mixed in equal amounts, and added to the fermentation broth.

### Experimental design

#### One-factor experimental design

**Fig. 1:**
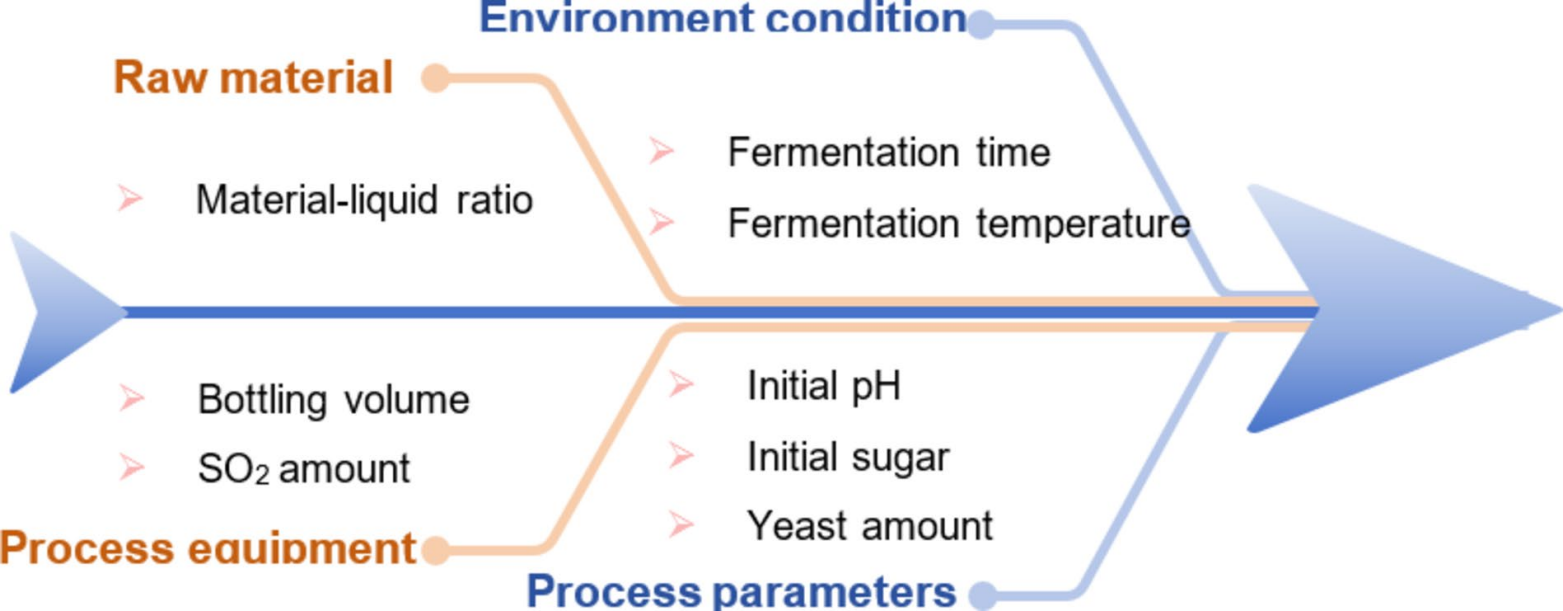
The potential factors influencing alcohol yield in the fermentation process.

The factors that may affect the results were fully considered. The potential factors influencing alcohol yield in the fermentation process was shown in Fig. 1. The alcohol content was accomplished as the main evaluation index, while the residual sugar and senses evaluation as the reference evaluation index. The concentration of the alcohol was determined via an alcohol meter and the residual sugar was determined using a DR201-95 digital refractor (A. KRÜSS OPTRONIC GMBH, Hamburg, Germany). PH was determined using a PP-20 pH meter (Sartorius, Krackeler Scientific Ltd., Albany, New York, USA). Sensory evaluation was performed using previous methods^2^. The influences of material-liquid ratio, initial pH, initial sugar, yeast amount, SO_2_ amount, fermentation temperature, fermentation time and bottling volume on the fermentation results were investigated respectively. The results of one-factor experimental obtained the optimal level of each factor for the subsequent experiments.

#### Experimental design of Plackett-Burman (PB), steepest climbing and Box-Behnken (BB)

The PB design was selected to investigate the 8 factors on the basis of one-factor design. The factor level with significant difference between the two ends of the optimal level in the single-factor experiment was taken as the + 1 and − 1 levels of the PB experiment, and alcohol content was taken as the response value. The data processing was performed to compare the F values and significance of each factor^27^.

The factors with significant response value were selected according to the results of PB experiment. The step direction and step size were set by the regression model and experimental experience, and six gradients were set, each gradient three parallel. The indexes were conducted according to the one-factor optimal conditions to determine the range and center points of significant factors in the subsequent experiments^27^.

According to the results of the PB experiment and the steepest climbing experiment, the BB experimental design was conducted based on the factors affecting the significance. The central point was the value of the corresponding factors of the highest alcohol content group in the steepest climbing experiment^27^.

### Optimization model establishment

#### RSM model establishment

RSM modeling was performed based on the mean value of the test data from the central combination design via Design-Expert software, and variance and regression analysis were performed. The following formula was obtained^28^.

$$\:Y={\beta\:}_{0}+\sum\:_{i=1}^{k}{\beta\:}_{i}{x}_{i}+\sum\:_{i=1}^{k}{\beta\:}_{ii}{x}_{i}^{2}\:+{\beta\:}_{ij}{x}_{i}{x}_{j}\:$$

Where: Y represents the response value of the equation. $\:{\beta\:}_{0}$ was a constant. $\:{\beta\:}_{i}$, $\:{\beta\:}_{ii}$ and $\:{\beta\:}_{ij}$ were the primary term coefficient, quadratic term coefficient and interaction term coefficient, respectively. $\:{x}_{i}$ and $\:{x}_{j}$ were the independent variable factors of the test (A, B, C and D).

#### Model establishment of ANN-GA

##### Artificial neural network (ANN) model establishment

**Fig. 2:**
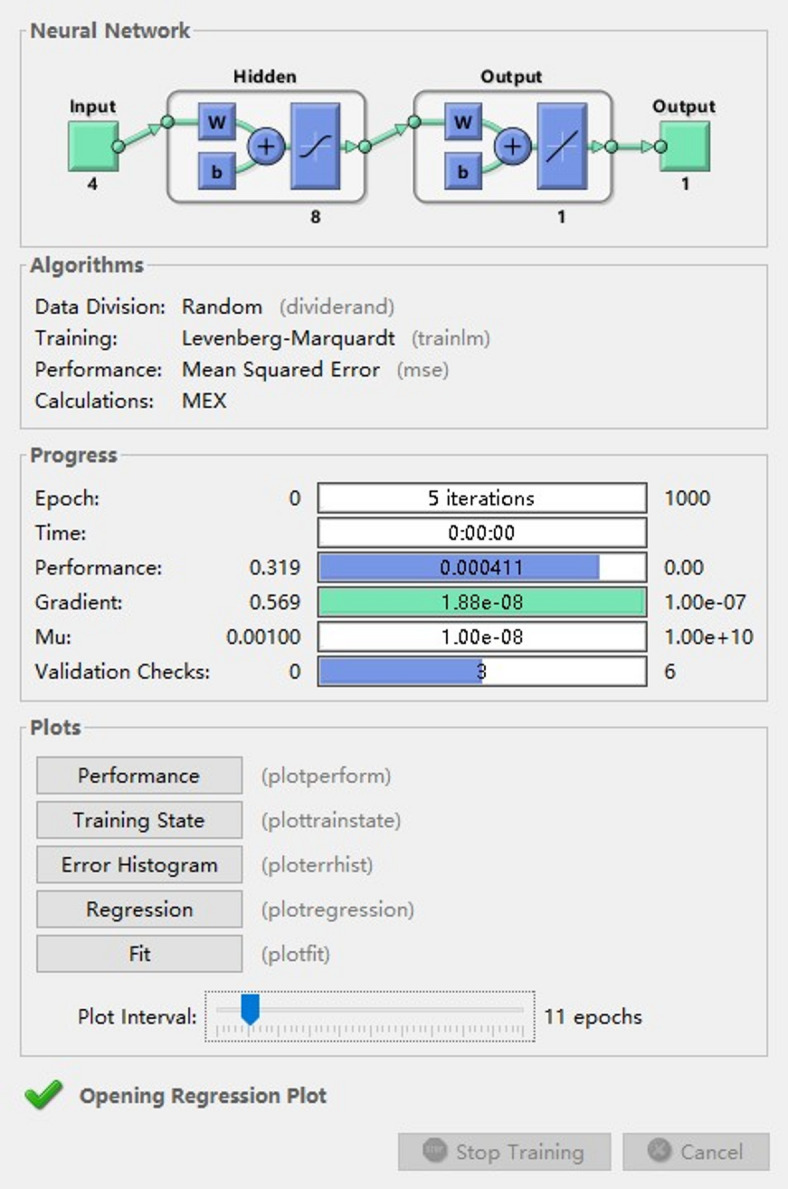
The modeling structure of ANN.

Twenty-nine samples were used to construct the ANN model, making the sample size consistent with the RSM model. A three-tier BP neural network was modeled using the Neutral Net Fitting toolbox in the Matlab R2021a software^23^. The modeling structure is shown in Fig. 2, which includes the input layer, hidden layer and output layer. Due to the non-uniformity of experimental data, the data should be normalized before data analysis. After the model training and prediction were completed, the Anti-normalization treatment was also carried out.

In order to improve the network training speed and reduce the prediction error, the ANN model was trained using the Levenberg-Marquardt algorithm, the Logsig function for the hidden layer and the Purelin function for the output layer. After the setting of the ANN model structure, we also need to set the parameters of the neural network, with a maximum number of iterations of 1000, an initial learning rate of 0.01, and an error threshold of 0.000001^24^.

Before optimizing the processing, the data were randomly disrupted, and divided according to a certain proportion into two parts: training set and test set. The training set accounted for 70%. The test set can be divided into two parts: validation data set and test data set, accounting for 15% respectively.

##### GA optimization based on ANN

The number of iterations, number of variables, crossover probability and variation probability also needed to be set before the genetic algorithm starts. Because the number of iterations and the number of variables directly affected the search accuracy and algorithm efficiency, the number of iterations was finally set to 50 times, the number of variables was 5, the parameters of the selection function was 0.09, and the crossover function parameter was 2. Optimize variable boundary matrix was − 1 to 1. The data was optimized using the goat toolbox of the genetic algorithm, and the algorithm was appropriately optimized to obtain the best optimization parameters^29^.

### Model validation and comparison

To test the validity and accuracy of the models, the difference between the actual values and predicted values of the optimized fermentation effect must be verified based on the RSM model and the ANN-GA model. The coefficient of determination (*R*^2^) and root mean square error (RMSE) were used to evaluate the predictive ability of the two models^20^. The specific formula was as follows:

$$\:{R}^{2}=1-\frac{\sum\:_{i=1}^{k}{X}_{i,p}-{X}_{i,e}}{\sum\:_{i=1}^{k}{X}_{i,e}-\stackrel{-}{X}}$$

Where: $\:{X}_{i,e}$ was the test value of group *i*, $\:{X}_{i,p}$ was the predicted value of group *i*, $\:\stackrel{-}{X}$ was the average value of all test values, and *k* was the number of test groups.

### Statistical analysis

All analysis were conducted in triplicate (*n* = 3), and values presented as mean ± standard deviation within the results tables. Duncan’s test of Analysis of Variance (ANOVA) was conducted using the SPSS 21.0 software package (IBM Corp, Armonk, America), with a significance level of *p* < 0.05 indicating statistical significance. The Plackett-Burman design and Box-Behnken design were completed by Design-Expert software (Version 13.0.1.0, Stat-Ease Inc., Minneapolis, MN, USA). The 3D modeling of the RSM data was also established by Design-Expert software (Version 13.0.1.0, Stat-Ease Inc., Minneapolis, MN, USA). The neural network data analysis and image rendering using Matlab software (Math work, USA, R2021a). Origin 2022 were used for other data processing and visualization.

## Results and discussions

### Results of the one-factor experiments

**Fig. 3:**
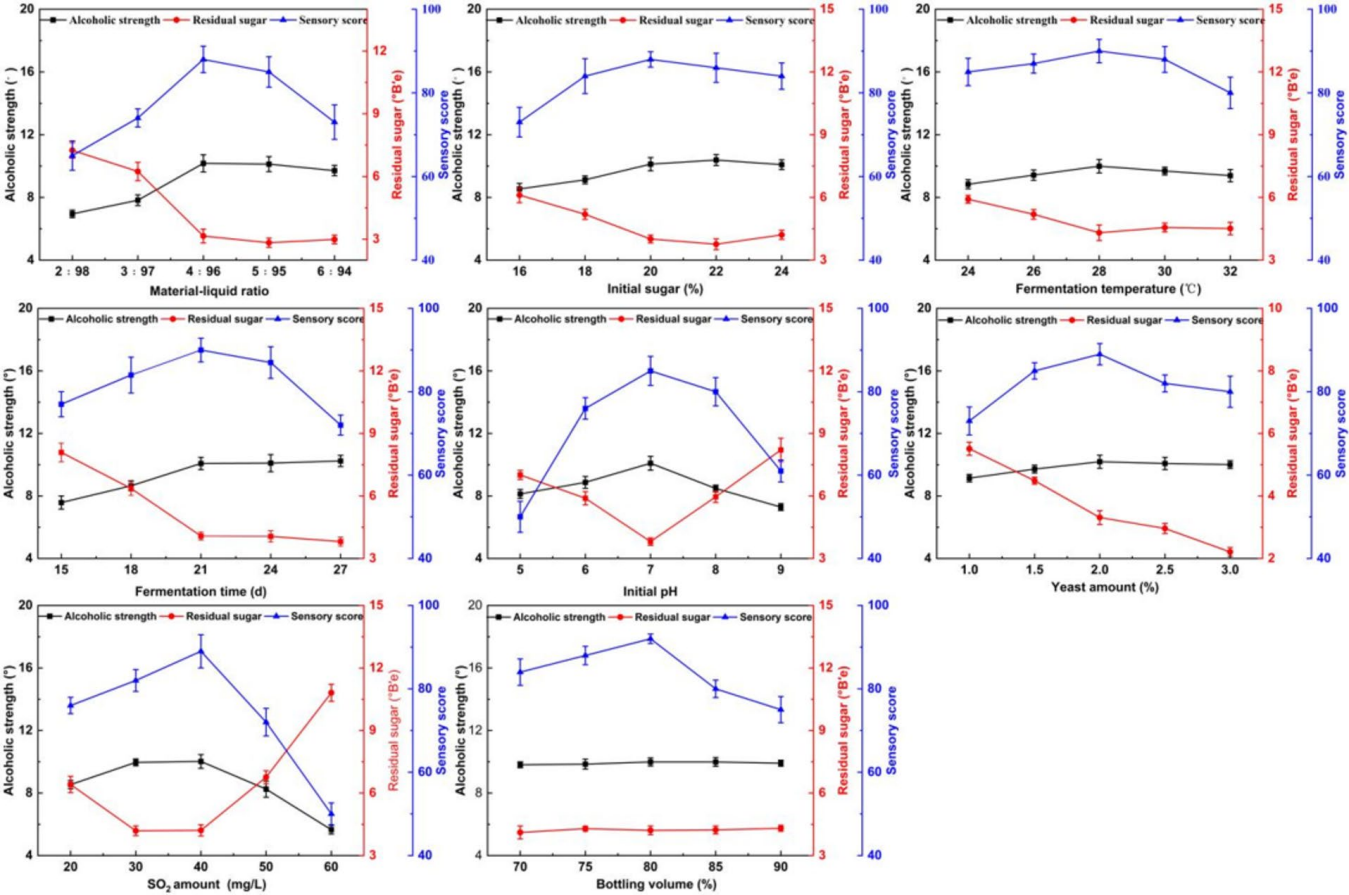
The results of one factor experiment.

The fermentation of fruit wine was a complex system, and the process was affected by many factors, so should be optimized^30^. The one-factor experiment was usually performed first to screen out the approximate range in which each factor has a significant effect on the fermentation results, and the results were shown in Fig. 3. The liquid ratio, initial sugar, yeast inoculum amount and SO_2_ amount were usually the key indicators considered, which were evenly reflected in many studies^31,32^. Other indicators (such as bottling volume etc. ) could also affect fermentation effect, which were usually considered the difference was not significant, so they were less used in studies^32^.

The alcohol content was used as the indicator of fermentation in this experiment, while residual sugar content and sensory score were only used as reference indicators and did not participate in the evaluation. We conducted the preliminary experiment, so the ethanol production of the fermentation factors was around the central point. In the one-factor experiments, only the initial pH and SO_2_ amount showed large fluctuations. Yeast fermentation itself would produce a certain acid, the fermentation process will be inhibited in the strong acid and strong alkali environment. Of course, high concentrations of SO_2_ amount could also inhibit yeast growth^33^. The residual sugar content and the ethanol amount had a certain corresponding relationship. There was also a little sugar loss in the fermentation process, which mainly provided energy for yeast growth^11^.

### Composite experimental design

#### The results analysis of the PB experiment

The Plackett-Burman experimental was 11 factors *n* = 12 (0 central values) and designed based on the results of the one-factor experimental. The experimental design and experimental results were shown in Supplementary File Table S1 and Table 1. The experimental values were the average value of three repeated experiments.

**Table 1:** Experimental design of PB and the results for production.

NO.	Factor								Yield (°)
	Material-liquid ratio (A)	Initial pH (B)	Initial sugar (C)	Yeast amount (D)	SO_2_ amount (E)	Fermentation temperature (F)	Fermentation time (G)	Bottling volume (H)	
1	−1	−1	−1	−1	−1	−1	−1	−1	7.48
2	−1	1	1	−1	1	1	1	−1	9.58
3	−1	−1	−1	1	−1	1	1	−1	8.45
4	1	1	1	−1	−1	−1	1	−1	9.82
5	1	−1	−1	−1	1	−1	1	1	8.96
6	−1	−1	1	−1	1	1	−1	1	10.19
7	1	−1	1	1	−1	1	1	1	10.45
8	1	1	−1	1	1	1	−1	−1	10.22
9	1	1	−1	−1	−1	1	−1	1	9.78
10	−1	1	1	1	−1	−1	−1	1	10.28
11	1	−1	1	1	1	−1	−1	−1	10.42
12	−1	1	−1	1	1	−1	1	1	9.58

The optimal regression equation was obtained through the analysis of the PB experimental results. R1 = 9.6008 + 0.3408 A + 0.2758B + 0.5225 C + 0.2991D + 0.2242E + 0.1775 F-0.1275G + 0.2725 H. To further analyze the effects of each factor on ethanol production, the experimental results were analyzed by regression variance (Table 2) and significant effects (Table 3).

**Table 2:** Regression equation analysis of variance in PB design.

Source	Sum of squares	Degree of freedom	Mean square	F value	*P* value
Model	8.72	8	1.09	18.85	0.0173
Residual	0.17	3	0.058		
Cor total	8.90	11			
R^2^	*R*^*2*^ = 0.9805				
Adjusted R^2^	*R*^*2*^_Adj_ = 0.9285				

The regression model had an F value of 18.85 and a corresponding P-value of 0.0173 (less than 0.05 and greater than 0.01), indicating that the model was significant. The correlation coefficient (0.9805) and the adjusted correlation coefficient (0.9285) were highly correlated between the predicted values and the experimental values, indicating that the model could well simulate the amount of ethanol generation, had a high degree of fit and a reliable experimental design.

**Table 3:** Partial regression coefficient and significance effect analysis in PB design.

Model item	Regression coefficient	Standard error	F-value	*P*-value	Significance
Intercept	9.60	—	—	—	
Material-liquid ratio	0.34	0.069	24.10	0.0162	*
Initial pH	0.28	0.069	15.78	0.0285	*
Initial sugar	0.52	0.069	56.63	0.0049	***
Yeast amount	0.30	0.069	18.56	0.0230	*
SO_2_ amount	0.22	0.069	10.42	0.0483	*
Fermentation temperature	0.18	0.069	6.54	0.0835	
Fermentation time	−0.13	0.069	3.37	0.1636	
Bottling volume	0.27	0.069	15.40	0.0294	*

***Means the difference was extremely significant (*P* < 0.01).

*Means the difference was significant (*P* < 0.05).

There were 6 factors with significant effects on ethanol production, namely solid-liquid ratio, initial pH, initial sugar, yeast amount, SO_2_ amount and bottling volume, among which the initial sugar had a very significant effect on ethanol production. To determine the fermentation effect more comprehensively, the above six significant factors were selected as the steepest climbing experimental factors.

#### The results analysis of steepest climbing experiment

The steepest climbing experiment was essential to assess the optimal level of the chosen factors after the screening procedure with PB based on statistical analysis^34^. According to the positive and negative effects of PB experiment and the results of one-factor experiment, the direction and step length of the steepest climbing experiment of each factor were determined. The experimental design and the results were shown in Table 4. The amount of ethanol production tends to increase first and then decrease, and the ethanol production was the highest. When the liquid ratio was 4.00 : 96.00, initial pH value was 7.00, initial sugar concentration was 22.00%, yeast amount was 2.00%, SO2 amount was 35.00 mg/L, the ethanol production was 10.20 mg/L, indicating that this condition was close to the optimal response area. Therefore, combined with the significance of factors in PB experiment, the factors (material-liquid ratio, initial pH, initial sugar and yeast amount) and their corresponding levels in group 4 were selected as factors and center points for the response surface experiment.

**Table 4:** Experimental design and the results of steepest ascent.

No.	Factor						Yield (°)
	Material-liquid ratio	Initial pH	Initial sugar (%)	Yeast amount (%)	SO_2_ amount (mg/L)	Bottling volume (%)	
1	2.5 : 97.5	5.50	20.50	1.25	20.00	65.00	8.74
2	3.0 : 97.0	6.00	21.00	1.50	25.00	70.00	9.51
3	3.5 : 96.5	6.50	21.50	1.75	30.00	75.00	10.20
4	4.0 : 96.0	7.00	22.00	2.00	35.00	80.00	10.25
5	4.5 : 94.5	7.50	22.50	2.25	40.00	85.00	10.03
6	5.0 : 95.0	8.00	23.00	2.50	45.00	90.00	9.66

#### The results analysis of center combination design

The center combination design (CCD) is a type of design of experiments consisting of a 2-level full factorial design with a center point and axial points^35^. And the CCD results allow a statistical estimation of linear and quadratic effects on a given response with a reduced number of experiments^36^. According to the CCD principle of four-factors and three-level, a total of 27 experimental points were setted up, among which the factorial part was 24 experimental points, and the repetition number of the central point was three times. The central combination design and resulting values are shown in Table S2 and Table 5.

**Table 5:** The experiment results of CCD.

NO.	Factor				Yield (°)
	A: Material−liquid ratio	B: Initial pH	C: Initial sugar	D: Yeast amount	
1	0	0	−1	−1	10.02
2	0	0	0	0	10.22
3	1	1	0	0	9.89
4	0	1	−1	0	9.88
5	−1	0	0	1	9.72
6	0	−1	−1	0	9.68
7	−1	1	0	0	9.81
8	−1	0	−1	0	9.61
9	1	0	0	1	10.18
10	0	−1	0	1	10.04
11	−1	0	0	−1	9.83
12	0	0	1	−1	10.21
13	0	1	0	1	9.99
14	1	−1	0	0	9.85
15	1	0	1	0	10.05
16	0	0	1	1	10.18
17	−1	−1	0	0	9.68
18	−1	0	1	0	9.71
19	0	1	1	0	10.06
20	0	0	−1	1	10.09
21	0	1	0	−1	9.95
22	0	0	0	0	10.11
23	1	0	0	−1	10.03
24	0	−1	0	−1	9.75
25	0	0	0	0	10.21
26	1	0	−1	0	9.87
27	0	−1	1	0	10.06

### Optimization process

#### Optimization analysis of the RSM

The experimental results were fitted to multiple quadratic regression using Design-Expert software and the analysis results were shown in Table 6. The resulting regression equation was as follows. *R*_1_ = 10.1800 + 0.1258 A + 0.0433B + 0.0933 C + 0.0342D-0.0225AB + 0.0200AC + 0.0650AD-0.0500BC-0.0625BD-0.0250CD-0.2338A^2^-0.1825B^2^-0.0850C^2^-0.0138D^2^. Primary terms A and C and interaction terms A^2^ and B^2^ reached extremely significant levels (*P* < 0.01), and interaction item C^2^ reached significant levels (*P* < 0.01). After excluding the insignificant term in the equation, the simplified regression equation was as follows. R1 = 10.1800 + 0.1258 A + 0.0933 C-0.2338A^2^-0.1825B^2^-0.0850C^2^.

**Table 6:** Regression equation analysis of variance of the CCD.

Source	Sum of squares	Degree of freedom	Mean square	F value	*P* value	Significance
Model	0.79	14	0.056	7.04	0.0008	***
A	0.19	1	0.19	23.73	0.0004	***
B	0.023	1	0.023	2.81	0.1193	
C	0.1	1	0.1	13.05	0.0036	***
D	0.014	1	0.014	1.75	0.2106	
AB	2.03E-03	1	2.03E-03	0.25	0.6242	
AC	1.60E-03	1	1.60E-03	0.2	0.6628	
AD	0.017	1	0.017	2.11	0.1719	
BC	0.01	1	0.01	1.25	0.2857	
BD	0.016	1	0.016	1.95	0.1877	
CD	2.50E-03	1	2.50E-03	0.31	0.5866	
A^2^	0.29	1	0.29	36.39	< 0.0001	***
B^2^	0.18	1	0.18	22.18	0.0005	***
C^2^	0.039	1	0.039	4.81	0.0487	*
D^2^	1.01E-03	1	1.01E-03	0.13	0.7289	
Residual error	0.096	12	8.01E-03			
Missing fit	0.089	10	8.87E-03	2.4	0.3301	
Pure error	7.40E-03	2	3.70E-03			
Correlation coefficient	*R*^2^ = 0.8914					
	*R*^2^_Adj_ = 0.7648					

***Means the difference is extremely significant (*P* < 0.01).

*Means the difference is significant (*P* < 0.05).

The regression model P value was 0.0008, while the mismatch term was 0.3301, which was a non-significant difference, indicating that the regression model fitted well and the regression equation could well describe the relationship between each factor and ethanol production^35^. The equation determination coefficient (0.8914) and the correction coefficient (0.7648) indicating that the second-order regression equation could explain the variability of the response surface, the experimental fit was good, the high correlation between the predicted value and the true value, the equation could be used to determine the best conditions for ethanol synthesis^30^.

**Fig. 4:**
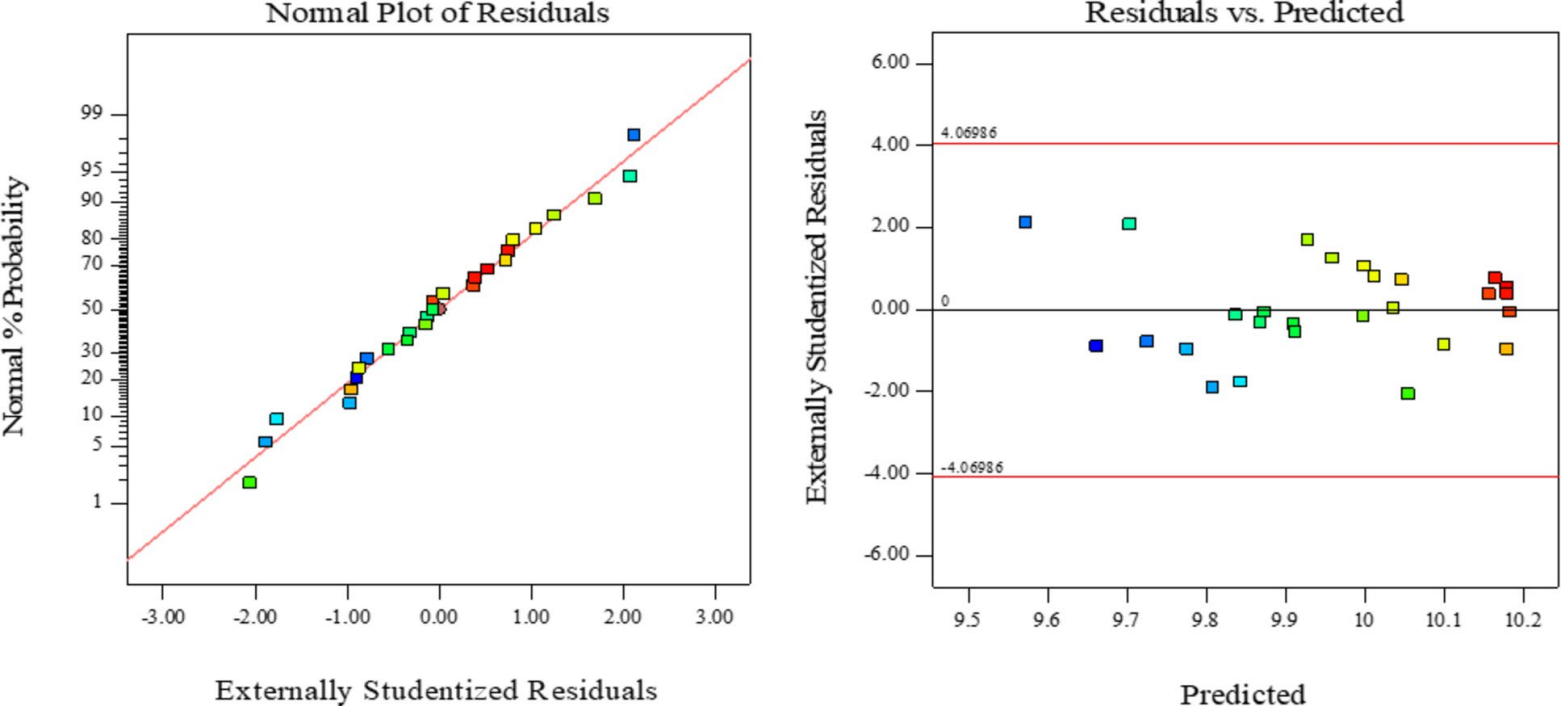
Model residual distribution diagram.

The simulation effect of the regression model could also be judged by using the residual distribution map of the model. Figure 4 were the distribution pattern of model residuals and the corresponding plots between model residuals and predicted values of the equation, respectively. The scoring points were distributed along the line, and most of the points were on the line, indicating that the residual was normally distributed; and the graph shown that the residual was within the prediction range and was scattered irregularly, indicating that the model fit is good^37^.

**Fig. 5:**
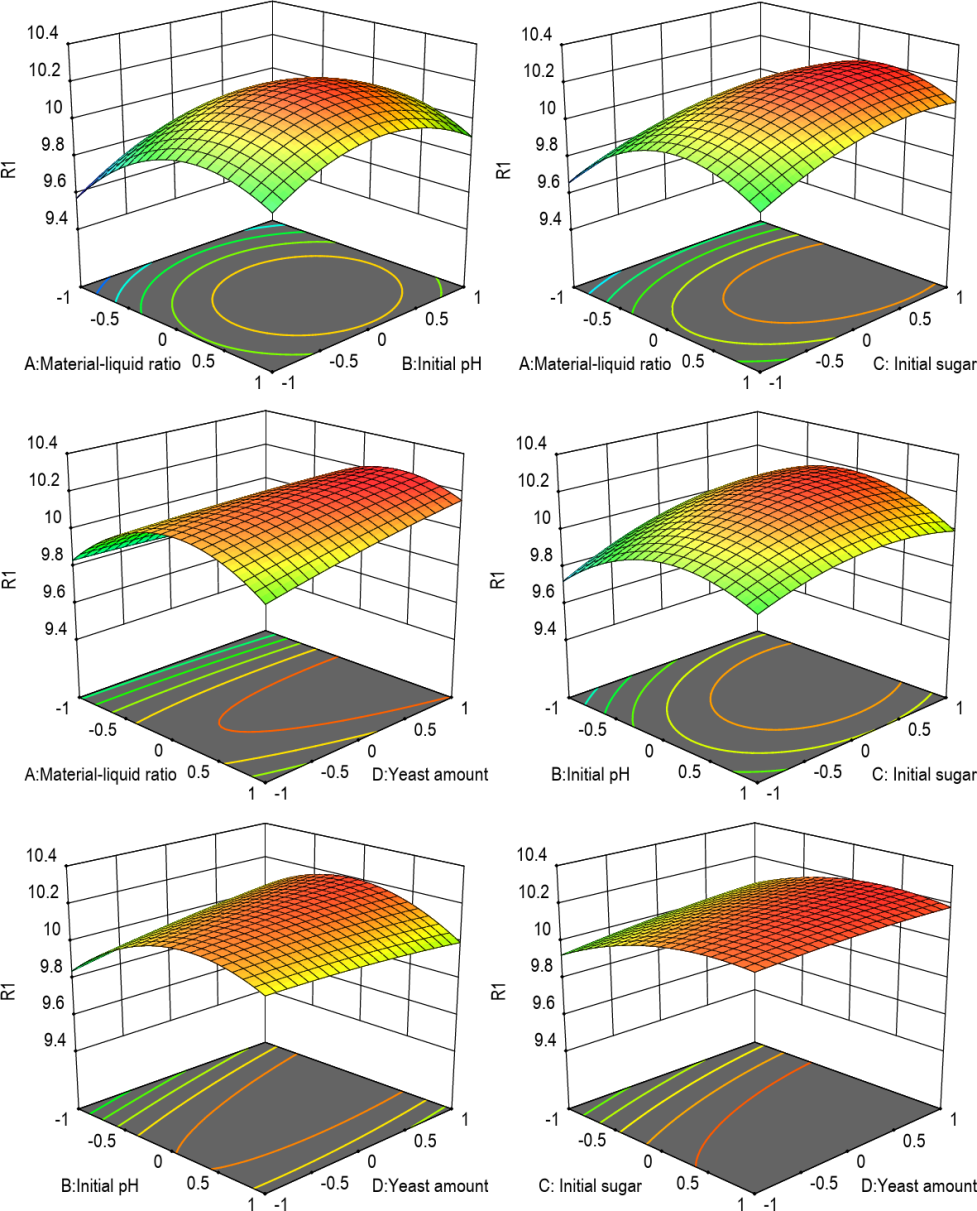
Response surface and contour map of the interaction of various factors on ethanol content.

The response surface and contour plots of the effect of each factor interaction on ethanol production are shown in Fig. 5. The steeper the slope of the response surface, the more sensitive the factor condition is to the yield change, the steeper the surface, the more sensitive the factor, and the less affected. The influence of each factor on ethanol production is: the liquid ratio > initial sugar concentration > initial pH > yeast addition; according to the regression equation, the influence of each factor on ethanol production, AD > BD > BC. The change trend of each factor index can be obtained by solving the regression equation. The best prediction conditions can be obtained: the liquid ratio is 4.2595.75, the initial pH value is 6.93, the initial sugar concentration is 22.250%, the yeast added amount is 2.00%, and the final predicted value was 10.260 mg/L.

#### Optimization analysis of ANN-GA

On the basis of the neural network framework, through the genetic algorithm, the fitness value decreases with the number of iterations, indicating that the genetic algorithm plays an obvious role in optimizing the initial weight of the network. After 27 iterations, the convergence state was reached, and the optimal fitness value was obtained. The fitness curves was shown in Figure S1. After the initial weight and threshold are optimized by the genetic algorithm, the fitness error drops below 0.075, the data was used as the initial weight and threshold of the neural network, and then the neural network was trained, that was, the optimized neural network prediction model was obtained^29^.

**Fig. 6:**
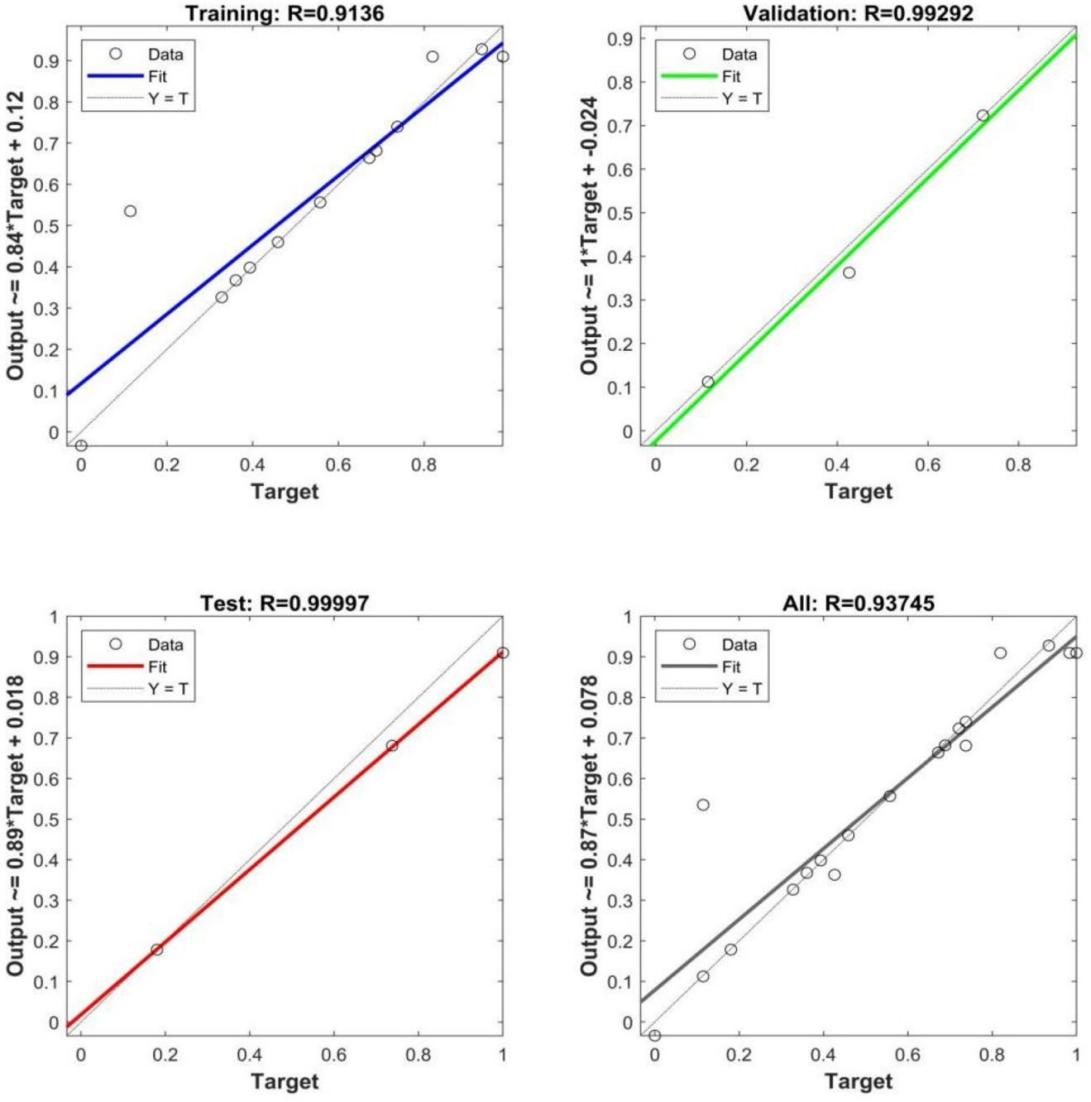
Simulation effect of ANN-GA.

The simulated effects of ANN-GA were shown in Fig. 6. When the network training times reaches the fifth time, the ANN-GA model achieves the best fitting effect within the preset parameter range, the training data set corresponds to the fitting R value was 0.9136, the validation data set corresponds to the fitting R value is 0.99292, the test data set was 0.99997, the corresponding fitting R value of all data was 0.93745, the fitting R value of each data set was close to 1, the model showed good simulation effect^21^.

**Fig. 7:**
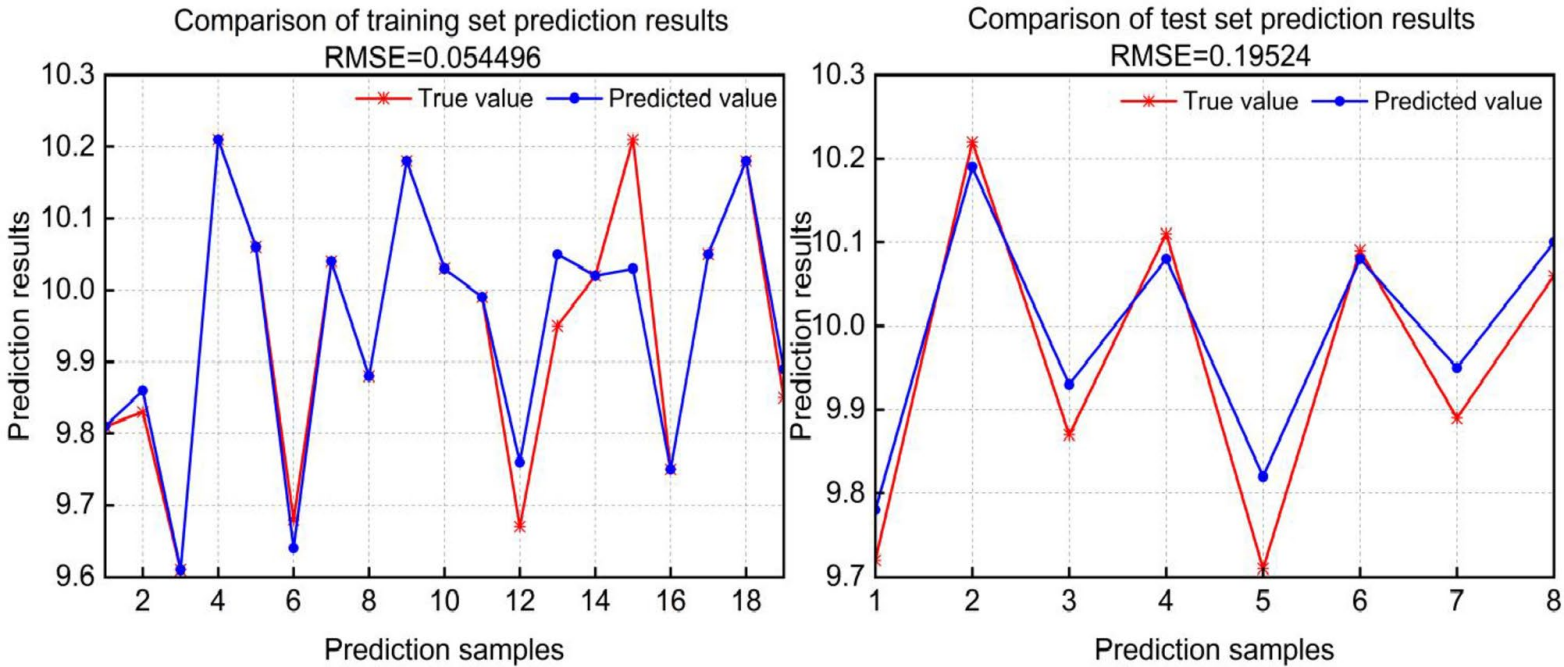
Effect comparison between the training set and the prediction set.

The effect comparison between the training set and the prediction set was shown in Fig. 7. The red color was the true value, and the blue color was the output value of the model. The output value was the same as the true value, showing the same curve trend on the coordinate axis. The error between the model output value and the true value was small, and the model training effect was good^38^. The model was optimized by the genetic algorithm, and the results showed that the material-liquid ratio was 4.25 : 95.75, the initial pH value was 6.92, the initial sugar concentration was 22.248%, the yeast addition was 1.98%, and the final predicted value was 10.255 mg/L. With the optimal value of the model, the verification value was basically consistent with the theoretical value, which shows that the accuracy of the neural network model was high and the simulation effect was good.

#### Comparison and verification between ANN-GA and RSM

**Fig. 8:**
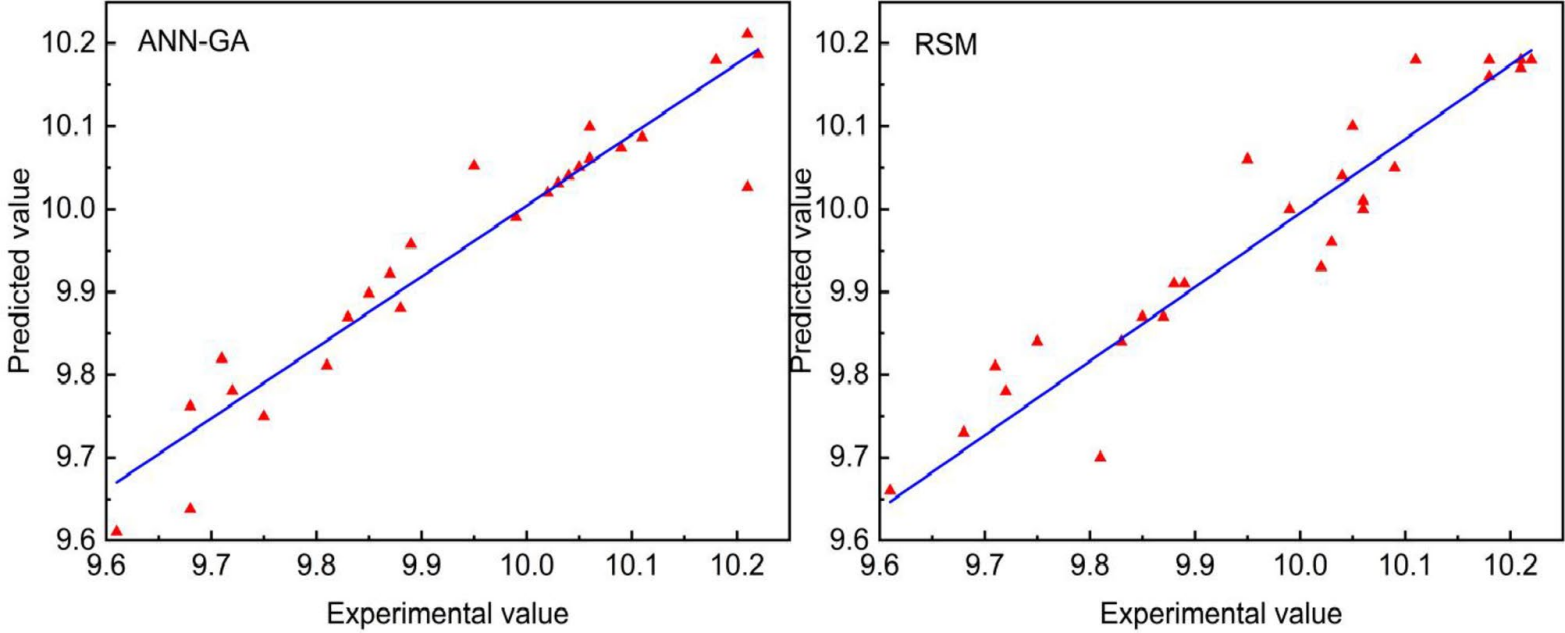
Comparison of optimization effect between ANN-GA and RSM.

The comparison between the predicted values and the experimental values under the respective optimal conditions is shown in Fig. 8. It can be seen that the lines drawn by the two are basically the same, but the prediction value of neural network optimization is slightly less than the response surface optimization method. The coefficient of determination R^2^ of the predicted value of the neural network was 0.9140, while the coefficient of determination R^2^ of the response surface was 0.8899. The RMSE of the predicted value of the neural network was 0.0896, the RMSE of the response surface was 0.0968. The fitting degree of the model was higher, and the prediction accuracy of the neural network fitting model was better^39^. The best fermentation process predicted by the ANN-GA was used as the experimental condition, and the finally resulted in a yield value was 10.248 mg/L. This had a better match with the predicted value.

## Conclusion

The fitting effect of the neural network model is better than the response surface model, and the fitting results are often more reliable. The reason may be that the neural network does not need the standard fitting function and infinite approximation ability when fitting the model. However, the neural network cannot explain the specific process of its reasoning, which is easy to fall into the local maximum when finding the extreme value, and the fitting process is prone to over fitting phenomenon. Therefore, so it is necessary to consider coupling analysis methods such as genetic algorithm to avoid the neural network modeling. Fermentation process optimization is essential in the research of alcohol fermentation, but its research technology is relatively mature. Generally, after single-factor uniform design, the response surface method is optimized. A similar design idea was adopted in this study, but the climbing design was further optimized, and the optimization effect of neural network model and response surface model was compared.

## Electronic supplementary material

Below is the link to the electronic supplementary material.

Supplementary Material 1

## Data Availability

Data is provided within the manuscript or supplementary information files.
