# Supplementary material for: Optimization process of coffee pulp wines combined with the artificial neural network and response surface methodology
Source: Sci Rep. 2025 May 14;15:16684. doi: 10.1038/s41598-025-00147-7 (PMC12078576; doi:10.1038/s41598-025-00147-7)
Supplement: Supplementary file 1 — Supplementary Material 1 [file 41598_2025_147_MOESM1_ESM.docx]

Supplemental instrument

Table S1 Factors and levels of the variables in PB design

| No. | Factor | Level | | |
| --- | --- | --- | --- | --- |
|  |  | -1 | 0 | +1 |
| A | Material-liquid ratio | 3﹕97 | 4﹕96 | 5﹕95 |
| B | Initial pH | 6.00 | 7.00 | 8.00 |
| C | Initial sugar | 20.00 | 22.00 | 24.00 |
| D | Yeast amount | 1.50 | 2.00 | 2.50 |
| E | SO_2_ amount | 30.00 | 40.00 | 50.00 |
| F | Fermentation temperature | 26.00 | 28.00 | 30.00 |
| G | Fermentation time | 18.00 | 21.00 | 24.00 |
| H | Bottling volume | 75.00 | 80.00 | 85.00 |

Table S2 Factors and levels of the BB experiment

| NO. | Factor | Level | | |
| --- | --- | --- | --- | --- |
|  |  | -1 | 0 | +1 |
| A | Material-liquid ratio | 3.50﹕96.50 | 4.00﹕96.00 | 4.50﹕95.50 |
| B | Initial pH | 6.50 | 7.00 | 7.50 |
| C | Initial sugar | 21.50 | 22.00 | 22.50 |
| D | Yeast amount | 1.75 | 2.00 | 2.25 |


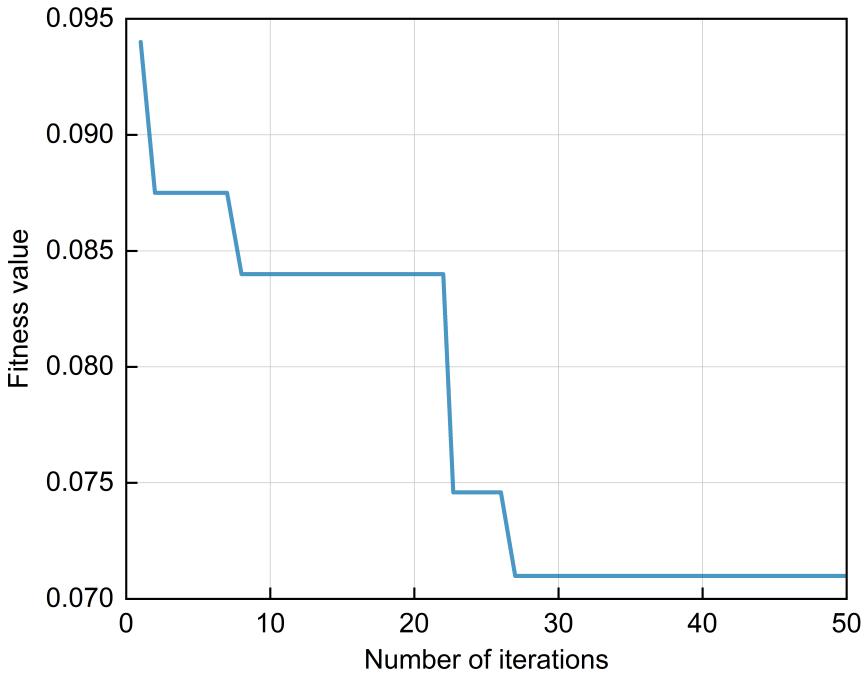


Fig. S1 Fitness curve of genetic algorithm
